# Will eye tracking change the way we diagnose and classify concussion and structural brain injury?

**DOI:** 10.2217/cnc.15.2

**Published:** 2015-08-06

**Authors:** Uzma Samadani

**Affiliations:** 1 New York Harbor HealthCare System, NY, USA; 2 Cohen Veterans Center for Post-Traumatic Stress & Traumatic Brain Injury; 3 Department of Neurosurgery, Psychiatry, Physiology & Neuroscience, New York University School of Medicine, 423 E, 23rd St, MC 112, Rm 4168N, New York, NY 10010, USA

**Keywords:** brain injury, concussion, eye tracking

Lee Schwamm’s recent editorial in the *New England Journal of Medicine* lamented the failure of the PROTECT III and SYNAPSE clinical trials after recruitment of more than 2000 brain injured patients, despite their being ‘exceptionally well designed and conducted’ [1]. Both of these trials selected and stratified patients on the basis of Glasgow Coma Scale (GCS) score and assessed outcome with extended Glasgow Outcome Scale Scores and secondary measures. Schwamm cited the lack of biomarkers for brain injury as one of the many causes of failure, and noted that "the investigators appropriately call for a comprehensive review of the TBI translational research strategy," particularly in light of the fact that these represent only the most recent of a long string of failed clinical trials for the treatment of brain injury [1].

Even before the failure of these most recent trials, recognition of the brain’s complexity and propensity for astonishing heterogeneity of injury has spurred a 22 country, 80 center effort in Europe. The Collaborative European NeuroTrauma Effectiveness Research in Traumatic Brain Injury trial is a prospective longitudinal observational study with one of its goals listed as improving ‘characterization and classification of TBI’ [2,3].

At a fundamental level, the mild/moderate/severe paradigm for brain injury most frequently based on GCS, loss of consciousness and post-traumatic amnesia has utility for triage and rapid communication during the acute phase of injury, but it is neither sufficiently sensitive to assess cognitive and neuropsychiatric deficits of TBI nor predictive of outcome [3]. Recently, I cared for a 44-year-old woman who had bumped her head at home, without any loss of consciousness or other symptoms whatsoever. She did not seek medical care. Two weeks later she had a headache and took aspirin. She developed a mild expressive aphasia and reported to her local ED, where a head CT revealed a subdural hemorrhage. This woman had a GCS that never slipped from 15. She never met the criteria for mild, moderate or severe TBI, yet could potentially have died without neurosurgical intervention and would likely have residual deficit requiring therapy.

Dogmatically, physicians will admit to the hospital or observe in the ED, any acute brain injury we can see on a CT scan, with the idea that these patients are at the highest risk for requiring surgical intervention. Equally dogmatically, we dismiss any patient without a visible structural brain injury on CT. And therein lies the problem. This subconscious allowance that the CT scan defines acute brain injury belittles the cryptic nature of concussive injury. The idea that something should be called ‘mild’ TBI, when it can potentially lead to a spiral of disabling and potentially lifelong symptoms, needs to be discarded.

The occurrence of neurologic injury in the absence of a blow to the head adds additional complexity to an already muddled picture. Patients with blast exposure and systemic impacts, whether through external shock waves, hypoxic effects or pressure gradients are now known to be vulnerable to a variety of cryptic neurologic disorders – some of which are exacerbated with time [4].

Without accurate diagnostics, classification schemes, outcome measures, and even a definition, the idea of estimating the incidence and impact of brain injury is a daunting one. It is hardly surprising that multiple clinical trials for therapeutics and prophylactics for brain injury result in successively more expensive failures.

Potential modalities for more accurate assessment and classification of brain injury include quantitative EEG, serum and other bodily fluid biomarkers, infrared spectroscopy and other tests.

Recently, there has been a renewed interest in eye tracking as a potential diagnostic, biomarker and outcome measure for brain injury, with our laboratory contributing to this effort.

The clinical basis for eye tracking as a diagnostic for brain injury has ancient roots. Some 3500 years ago, Egyptian physicians wrote the oldest known surviving surgical treatise stating that eyes that are askew may be evidence of brain injury [5,6]. Prior to the invention of radiographic imaging, the assessment of eye movements was a major modality of diagnosis of neurologic impairment with entire textbooks dedicated to this topic.

Modern era optometrists can detect abnormal eye movements in up to 90% of patients with so-called mild traumatic brain injury or concussion [7–12]. The most commonly detected abnormal eye movement associated with brain injury is a vergence problem [7]. Vergence is the ability of the both eyes to focus together on a single point. If the point moves closer to the nose, the pupils converge. Following the point in space – or while watching TV – requires sustained vergence. Previous studies using eye tracking to assess patients with postconcussive symptoms suggest that these deficits may persist beyond the acute phase of injury [13–15].

We have recently published two papers on eye tracking. In the first, we demonstrate that our novel tracking algorithm detects palsies of cranial nerves III and VI. It also detects mass effect near those nerves and the eye tracking changes caused by that mass effect are reversed by surgical relief of that mass effect [16].

The second paper recruits 75 trauma patients divided into three groups and compares them to 69 uninjured controls [17]. Group one hit their heads and had injury apparent on brain imaging. Group two hit their heads and had no injury apparent on imaging, and group three were trauma patients with other injuries not requiring brain imaging at all. Patients who hit their heads (groups one and two) had eye movements that were not as coordinated as uninjured healthy controls, while patients who did not hit their heads (group three) had eye movements that were as coordinated as uninjured healthy controls. Spearman correlation demonstrates that the severity of eye movement dysfunction correlates with the severity of concussion symptoms in all trauma subjects. Patients who had hit their heads were found to be worse in the 1–2 weeks following injury and then gradually recovered towards normal within the next month [17].

In this issue of *Concussion*, we determine the sensitivity and specificity of classifying schemes based on eye tracking as a biomarker for brain injury. Because there is currently no gold standard diagnostic for concussion to use as a true positive, we have relied on SCAT3, the most validated concussion measure in the literature. We used two different SCAT3 subsets, the symptom severity score and standardized assessment of concussion to identify true positive concussions within a group of trauma patients. Our results suggest a high correlation between these SCAT3 subsets and abnormal eye tracking metrics. An additional major finding of this paper is that approximately 70% of the patients who are evaluated in the ED and undergo brain imaging have physiologically normal brain function, as assessed by eye tracking. This finding would suggest that most people hit in the head do not have a brain injury and may potentially have headache, nausea, vomiting or dizziness from scalp, neck, inner ear, pain or visceral causes.

Pending papers from our laboratory demonstrate a correlation between elevated ICP and abnormal tracking, assess the impact of alcohol, narcotics and strabismus on tracking, and demonstrate how supra- and infranuclear lesions can be localized.

These recent studies raise the question of how eye tracking may change the way we diagnose and classify brain injury. We will consider these questions separately.

With regard to diagnosis, former Sun Microsystems CEO and venture capitalist Vinod Khosla among others has famously argued that objective measures and data will drive diagnostics in healthcare, as they are less subject to human interpretation and bias. He states that from a medicolegal perspective, this is inevitable. With our system, after getting hit on the head or subjected to impactful or blast trauma, a person simply watches television while getting eye tracked for 220 s. The assessment of their eye movements reveals whether there is: acute mass effect; elevated intracranial pressure; and disruption of pathways controlling ocular motility. Thus, we can rapidly, noninvasively and objectively quantitate the extent of physiologic impairment from brain injury.

Objective measures of radiographically silent neurologic disruption will enable more precise definition of the word concussion. Currently, there are more than 42 organizations that have posited definitions of concussion. Many of these definitions encompass any symptoms related to scalp, neck, inner ear or other systemic problems, in addition to brain injury. I would argue that eye tracking might ultimately be used to classify – or even define concussion – and limit its scope to traumatic neurologic injury not apparent on CT scanning and resulting in intracranial mass effect, elevated intracranial pressure or disruption of neurologic pathways.

The implications of a test that distinguishes patients with concussion caused by actual brain injury from those who have trauma to the scalp, neck, inner ear or systemics include improved classification of subject groups entering clinical trials and the ability to assess outcomes from these trials quantitatively. Similarly, improved classification of patients with structural brain injury enrolling in trials is also enabled with eye tracking. Therapeutics and prophylactics for both structural brain injury and concussion can then be developed, with hopefully greater success than in the past.
